# The Association of Anesthesia Type and Neonatal Outcomes Following Category-1 Cesarean Delivery: A Retrospective Cohort Study

**DOI:** 10.7759/cureus.35910

**Published:** 2023-03-08

**Authors:** Carl M Skoog, Joel F Katzer, Linder H Wendt, Unyime Ituk

**Affiliations:** 1 Carver College of Medicine, University of Iowa, Iowa City, USA; 2 Institute of Clinical and Translational Research, University of Iowa, Iowa City, USA; 3 Department of Anesthesia, University of Iowa, Iowa CIty, USA

**Keywords:** apgar score, umbilical arterial ph, epidural anesthesia, general anesthesia, cesarean delivery

## Abstract

Objectives

Neuraxial anesthesia is the preferred anesthesia technique for cesarean delivery due to a decreased risk of adverse events. However, general anesthesia is often employed during emergent cesarean deliveries to achieve a shorter decision-to-delivery interval. The objective of this study was to determine if the conversion of epidural labor analgesia to surgical anesthesia for a category-1 cesarean delivery is associated with significant neonatal morbidity.

Study design

This was a retrospective cohort study of all intrapartum category-1 cesarean deliveries performed at an academic tertiary care institution between August 2016 and July 2021. The primary outcome was neonatal morbidity, defined as a composite of neonatal umbilical artery pH < 7.10 and/or 5‐min Apgar score < 7, and/or neonatal intensive care unit admission. A multivariate regression analysis was performed to control for the presence of covariates and examine the degree to which they influenced the outcome.

Results

A total of 185 mother-neonate pairs qualified for inclusion, of which 23 had cesarean delivery under general anesthesia and 162 under epidural anesthesia. There was no significant difference in adverse neonatal outcomes between category-1 cesarean deliveries done under general anesthesia compared to epidural anesthesia (47% vs 35%, p = 0.3). The incidence of umbilical arterial pH < 7.10 was higher in the general anesthesia group compared to the epidural anesthesia group (35% vs 12%, p = 0.018). The multivariate regression model showed that gestational age (OR = 0.63; 95% CI = 0.51-0.75, p = <0.001) and non-reassuring fetal heart trace (OR = 0.18; 95% CI = 0.05-0.58, p = 0.005) were significant predictors of adverse neonatal outcome.

Conclusion

Our results suggest that the conversion of epidural analgesia to surgical anesthesia for category-1 cesarean delivery in women with a functional labor epidural catheter is not associated with poorer neonatal outcomes.

## Introduction

Emergency cesarean delivery (CD) is required when there is an immediate threat to the life of the fetus or mother. To expedite delivery, there must be clear, unambiguous communication between the obstetric, nursing, and anesthesia teams caring for the patient. In 2016, the obstetric unit at our institution adopted the classification system for the urgency of CD first proposed by Lucas et al. and standardized by the Royal College of Obstetricians and Gynaecologists (RCOG) in the United Kingdom [1,2]. The classification system grades the urgency of CD from category-1 (emergent) to category 4 (scheduled elective) CD. For a category-1 CD, a 30-minute decision-to-delivery interval (DDI) is recommended by various professional societies and used as a benchmark of the performance of an obstetric unit by the National Institute for Clinical Excellence (NICE) in the United Kingdom [3]. The timely initiation of surgical anesthesia plays a vital role in achieving the DDI goal. Therefore, it is not uncommon for obstetricians to express a preference for induction of general anesthesia (GA), even in parturients with indwelling functional labor epidural catheters. This is based on the assumption that the time required to convert epidural labor analgesia to surgical anesthesia will significantly prolong the DDI and potentially worsen outcomes for either the fetus or the mother [4]. Although GA reduces induction to incision time, parturients have an increased risk of difficult intubation and failed intubation [5]. GA is also associated with an increased risk of pulmonary aspiration, intraoperative awareness, postpartum hemorrhage, lower umbilical arterial pH compared to other anesthetic techniques, and an increased transfer of anesthesia drugs from the mother to the fetus [4,6-8]. Furthermore, GA does not allow for maternal participation in the birthing process or early skin-to-skin contact, which is associated with successful breastfeeding initiation and decreased maternal anxiety and depression [9-11]. The main objective of this study was to determine if the conversion of epidural labor analgesia to surgical anesthesia for a category-1 CD is associated with significant neonatal morbidity compared to when done under GA. We hypothesized that topping up the epidural catheter for intrapartum category-1 CD in women with adequate labor epidural analgesia is not associated with poorer neonatal outcomes.

## Materials and methods

Study design

This was a retrospective cohort study of all intrapartum category-1 cesarean deliveries performed at the University of Iowa Hospitals and Clinics between August 2016 and July 2021. The study was approved by the University of Iowa Institutional Review Board (IRB # 201911151), and a waiver of consent was obtained before data collection. The electronic medical record was queried for all CDs during the study period, and individual chart reviews were performed to determine study eligibility. Study data were collected and managed using Research Electronic Data Capture (REDCap) tools hosted at the University of Iowa. We included all CDs classified as category-1 in which epidural analgesia was established in labor before the decision to deliver emergently. Cesarean deliveries were excluded if there was documentation of downgrading of urgency after the patient's arrival in the operating room. To determine if the urgency of delivery was downgraded before the surgical incision, we utilized the operating room entry to skin incision interval (ORII) as a reference. Operating room-to-skin incision interval (ORII) served as the most reliable way to determine the acuity of urgent delivery, as a valid category-1 CD would have a short ORII. Cases with ORII of > 10 minutes were deemed to have been downgraded in the level of urgency and excluded from the analysis. A previous study reported a significant effect of the urgency of CD on the ORII and has been suggested as an accurate surrogate for the urgency of delivery [12]. The parturients were divided into two groups: the epidural anesthesia (EA) group and the GA group. Parturients who received an epidural top-up for surgical anesthesia but later converted to GA due to inadequate sensory block during surgery were in the EA group. Data extracted from the electronic medical records included maternal demographics, intraoperative blood pressure, anesthesia technique, drugs administered, the decision to delivery interval (DDI), ORII, skin incision to hysterotomy interval, hysterotomy to delivery interval, the total dose of vasopressors administered before delivery, indication for CD, Apgar scores at 1 and 5 minutes, umbilical arterial pH, base deficit, birth weight, neonatal intensive care unit (NICU) admission, and NICU length of stay. Umbilical blood samples for acid-base analysis are routinely collected immediately after delivery for all CDs at our institution. For the umbilical arterial pH to be valid and included in the data set, the value had to be less than the venous pH and the partial pressure of carbon dioxide higher than the partial pressure of the venous carbon dioxide. 

Management of labor analgesia and anesthesia for category-1 CD

In our obstetric unit, we offer a combined spinal epidural (CSE) analgesia technique for labor analgesia unless contraindicated. When the obstetric team decides to perform a category-1 CD, it is communicated via the emergency paging system, and the patient is immediately transferred to the operating room. In women with functional epidural catheters, a bolus of 3% Chloroprocaine 20 mL is administered via the epidural catheter on route to or on entry to the operating room. Alternatively, a mixture of 2% Lidocaine with epinephrine 1:200,000 is administered in up to four 5 mL aliquots for a total bolus dose of 20 mL. Hemodynamic monitoring is established, and the abdomen is prepared and draped. Before a surgical incision is made, the sensory block level is assessed. If a sensory block to the T4 dermatome is not achieved, rapid sequence induction of GA with propofol (2-2.5 mg/kg) and succinylcholine (1 mg/kg) is performed. However, depending on the scenario or following specific requests from the obstetrician, GA may be induced without attempting to top up the epidural catheter. Non-invasive blood pressure (NIBP) measurements are recorded at 1-minute intervals once the epidural top-up is given, or GA is induced until delivery and every 2.5 minutes subsequently. A prophylactic phenylephrine infusion is administered at a rate of 0.25-0.75 mcg/kg/min (total body weight) for the management of hypotension.

Statistical analysis

Descriptive statistics were used to summarize demographic characteristics and neonatal outcome variables stratified by anesthesia type. Categorical measures are reported as counts and percentages. Continuous measure distributions were assessed for normality using the Shapiro-Wilk test. Normally and non-normally distributed measures are reported as means and standard deviations or medians and interquartile ranges, respectively. Tests for differences between the anesthesia types were performed using Wilcoxon rank sum tests for continuous measures and Fisher’s Exact Test for categorical measures. The primary outcome for this study was neonatal morbidity, defined as a composite of umbilical artery pH <7.10 and/or 5‐min Apgar score <7, and/or NICU admission. A multivariate regression analysis was performed to control for the presence of other covariates and examine the degree to which they influenced the outcome. This model was initially fit with anesthesia type, ORII, and the interaction between these two variables as the predictors. Then, all variables from the candidate pool of additional variables were investigated, and the variable that most greatly reduced the Akaike information criterion (AIC) was added to the model. This process was repeated until no more additions from the candidate pool of covariates reduced the model’s AIC. The candidate pool of additional covariates consisted of gestational age, birth weight, induction of anesthesia to delivery time, maximum drop in systolic blood pressure, and indications for CD. Any candidate variables that caused convergence issues with the model due to their low prevalence in our study were excluded from consideration for the multivariate model. All logistic modeling is reported as odds ratio estimates, 95% confidence intervals, and p-values for all covariates. All tests were conducted using an alpha = 0.05 significance level. Univariate logistic models assessing the impact of anesthesia type were constructed for each of our secondary outcomes of interest, which include umbilical cord arterial pH, umbilical cord arterial base deficit, NICU length of stay, DDI, and ORII. For all binary outcomes, receiver operator characteristic (ROC) curves were generated to assess the relationship between the ORII and each of the outcomes of interest. Optimal cutoff value, sensitivity, specificity, and area under the curve (AUC) were recorded for each outcome. A sensitivity analysis was performed excluding patients that had a conversion of EA to GA. This was done to confirm that this subgroup of patients was not driving the findings of our primary analysis.

## Results

**Figure 1:**
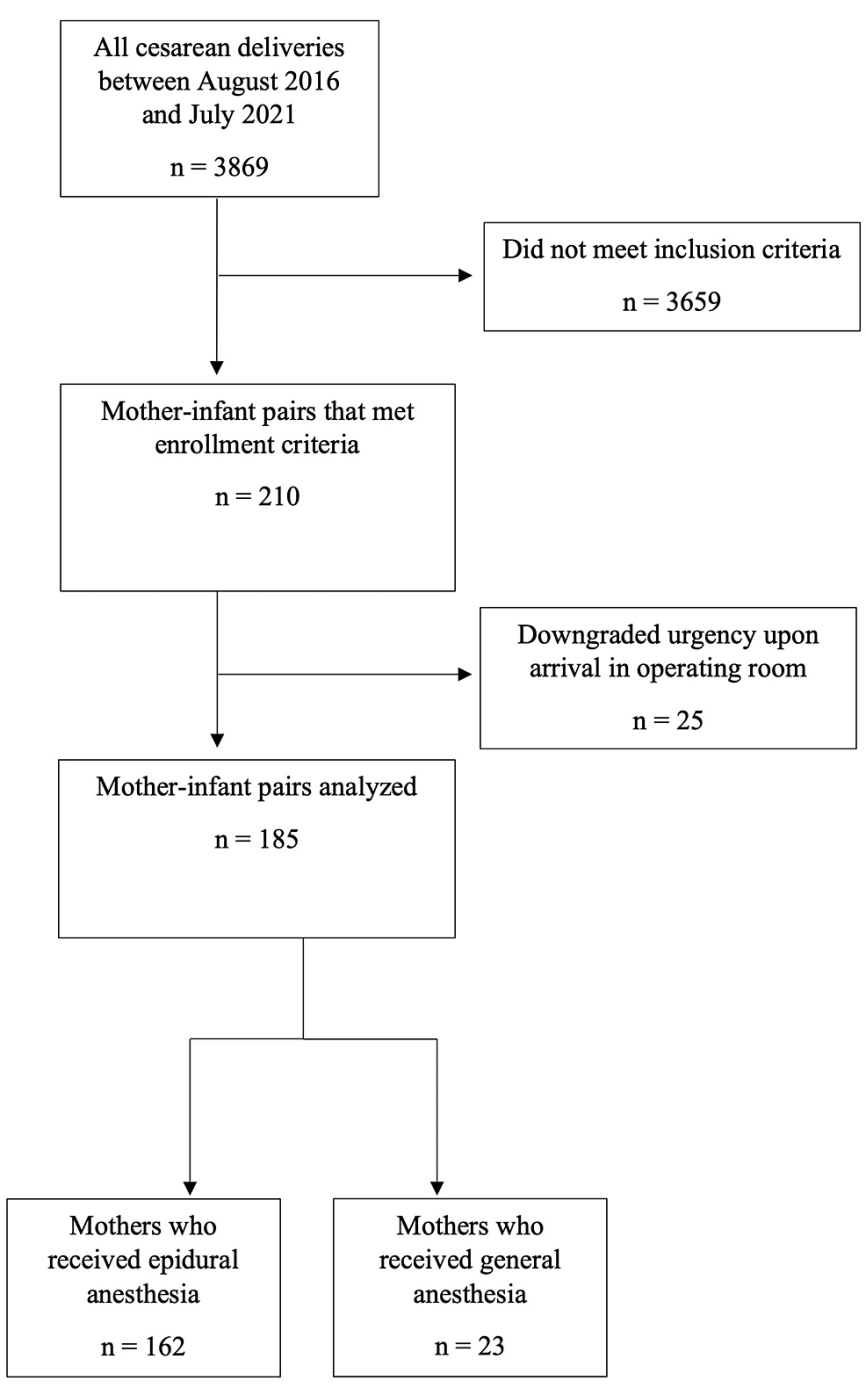
Flow diagram of subject identification and inclusion in the study

A total of 3,869 cesarean deliveries were performed between August 2016 and July 2021, accounting for 32.6% of all deliveries. Two hundred and ten mother-neonate pairs met enrollment criteria. We excluded 25 women because the urgency of CD was downgraded on arrival in the operating room. A total of 185 mother-neonate pairs; EA group =162 and GA group = 23 were included in the final analysis (Figure 1). Fifty-two women in the EA group required conversion from EA to GA before delivery of the neonate. There was no difference in maternal demographics and gestational age at delivery. However, there were significant differences in the indications for CD between the groups (Table 1). There was no significant difference in the incidence of composite adverse neonatal outcomes between category-1 CDs done under GA versus EA (47% vs 35%, p = 0.3). Umbilical arterial blood gas results were unavailable in six women in the GA group and 25 women in the EA group. The incidence of umbilical arterial pH < 7.10 was higher in the GA group compared to the EA group (35% vs 12%, p = 0.018). Neonatal outcomes are summarized in Table 2. EA was associated with a longer DDI, ORII, and induction of anesthesia to delivery time compared to GA. The relationship between measured time intervals and anesthesia technique is summarized in Table 3.

**Table 1:** Parturient and obstetric characteristics of category-1 cesarean deliveries Mean (SD); Median {IQR}; BMI, body mass index; AFE, amniotic fluid embolism; CD, cesarean delivery; GA, gestational age

	Overall, N=185	GA, N = 23	Epidural, N = 162	P-value
Age (years)	29 (6)	29 (7)	29 (6)	>0.9
BMI (kg/m^2^)	33 {29, 40}	33 {28, 39}	33 {29, 40}	0.7
GA weeks	39 {37, 40}	39 {37, 40}	39 {37, 40}	>0.9
Primiparous	90 (49%)	10 (43%)	80 (49%)	0.6
Previous CD	35 (19%)	4 (17%)	31 (19%)	0.84
Indications for CD				
NRFHT	156 (84%)	14 (61%)	142 (87%)	<0.001
Failed vacuum or forceps delivery	2 (1%)	0	2 (1%)	0.59
Cord prolapse	16 (8.6%)	5 (22%)	11 (6.8%)	0.033
Placental abruption	3 (1%)	1 (4%)	2 (1%)	0.26
Uterine rupture	6 (3.2%)	1 (4.3%)	5 (3.1%)	0.6
AFE	2 (1.1%)	2 (8.7%)		0.015
Phenylephrine dose (mg)	1.83 {0.14, 3.10}	1.26 {0.00, 3.76}	1.87 {0.20, 2.97}	0.5

**Table 2:** Neonatal outcomes for category-1 cesarean delivery Median (IQR); n (%); UA, umbilical artery; UABD, umbilical artery base deficit; NICU, neonatal intensive care unit; LOS, length of stay

	Overall, N = 185	GA, N = 23	Epidural, N = 162	P-value
Birth weight (kg)	3.15 {2.79, 3.55}	3.31 {2.78, 3.61}	3.14 {2.79, 3.53}	0.7
UA pH	7.21 {7.13, 7.27}	7.17 {7.09, 7.22}	7.21 {7.15, 7.27}	0.03
Apgar < 7 @ 1min	80/185 (43)	17/23 (74)	63/162 (39)	0.002
UABD >12 (mmol)	8/154 (5.2)	2/17 (12)	6/137 (4.4)	0.2
All composite outcome	5/154 (3.2)	3/17 (18)	2/137 (1.5)	0.01
Composite outcome items				
UA pH <7.1	22/154 (14)	6/17 (35)	16/137 (12)	0.02
Apgar < 7 @ 5min	16/185 (8.6)	4/23 (17)	12/162 (7.4)	0.12
NICU Admission	45/185 (24)	7/23 (30)	38/162 (23)	0.5
NICU LOS	15 {7, 36}	9 {4, 12}	23 {7, 39}	0.05

**Table 3:** Anesthesia technique and time intervals during category-1 cesarean delivery Median (IQR); DDI, decision to delivery interval; ORII, operating room entry to skin incision interval

	GA, N = 23	Epidural, N = 162	P-value
DDI (min)	13 (8, 17)	17 (12, 30)	0.014
ORII (min)	5 (4, 6)	8 (5, 14)	0.002
Skin Incision to Hysterotomy (min)	1.00 (0.00, 1.00)	2.00 (1.00, 3.00)	0.044
Hysterotomy to Delivery (min)	1.00 (0.00, 1.25)	1.00 (1.00, 1.00)	0.4
Induction to delivery time (min)	3 (2, 4)	8 (4, 12)	0.002

In the unadjusted regression model, there was no difference in the composite outcome of umbilical artery pH <7.10 and/or 5-min Apgar score <7, and/or NICU admission in the EA group compared to the GA group (OR=0.60; 95% CI=0.23-1.26, p =0.3). The multivariate regression model showed that gestational age (OR = 0.63; 95% CI= 0.51-0.75, p = <0.001) and NRFHT (OR = 0.18; 95% CI= 0.05-0.58, p = 0.005) were significant predictors of the composite adverse neonatal outcome. The summary of the multivariate regression model is shown in Table 4. A sensitivity analysis excluding patients who had a conversion from EA to GA yielded similar results. A receiver operator characteristic curve assessing the relationship between ORII and the composite outcomes showed that ORII was of minimal predictive value (area under the curve, AUC = 0.5).

**Table 4:** Multivariate regression for the composite neonatal outcome OR, odds ratio; CI, confidence interval; GA, general anesthesia; EA, epidural anesthesia; ORII, operating room entry to skin incision interval; NRFHT, non-reassuring fetal heart trace

Characteristic	OR	95% CI	P-value
GA	-	-	
EA	1.79	0.28, 23.5	0.4
ORII	1.18	0.94, 1.87	0.2
Gestational age (weeks)	0.63	0.51, 0.74	< 0.001
NRFHT	0.18	0.05, 0.58	0.005
EA * ORII	0.82	0.52, 1.04	0.3

## Discussion

The result of our study indicates that the conversion of epidural labor analgesia to surgical anesthesia for category-1 CD in women with functional labor epidural catheters is not associated with poor neonatal outcomes. The multivariate regression model indicated that after adjusting for the longer ORII and DDI associated with EA, the gestational age of the newborn and non-reassuring fetal heart trace (NRFHT) were the only significant predictors of neonatal morbidity. 

Several studies have investigated the effect of anesthesia techniques for emergent CD on neonatal outcomes. A study conducted at a tertiary care obstetric unit in France reported that GA was associated with an increased incidence of neonatal respiratory interventions, transfer to the neonatal intensive care unit, and neonatal mortality [13]. A similar 2021 study by Metogo et al. found that GA was associated with lower APGAR scores at 1 and 3 minutes as well as a higher incidence of neonatal resuscitation [14]. However, it is still generally accepted that GA allows for a shorter DDI, which is desirable for category-1 CD. Unlike previous studies, we chose to study only parturients with indwelling epidural catheters who presented for category-1 CD to determine if the longer DDI does influence neonatal outcomes. A recent study in the National Health Service (NHS) in England by Bhatia et al. reported a concurrent increase in neuraxial anesthesia utilization for category-1 CD and DDI during the COVID-19 pandemic [15]. The study also noted that the incidence of adverse neonatal outcomes was similar between the pre-COVID-19 and post-COVID-19 groups, suggesting that the increase in DDI with neuraxial anesthesia use does not adversely affect neonatal outcomes. 

Our data showed that cord prolapse was the indication for CD more likely to be performed under GA. This is not unexpected considering that a GA is associated with shorter DDI and the need for immediate delivery. However, a significant number of CDs performed for cord prolapse in our cohort were done by topping up the indwelling labor epidural catheter to achieve surgical anesthesia. Therefore, it is reasonable to attempt using neuraxial anesthesia for CD in women with functional indwelling labor epidural analgesia catheters, even in these critical scenarios. There is concern that attempting neuraxial anesthesia for category-1CD and subsequently converting to GA if unsuccessful may be associated with a higher risk of neonatal morbidity versus proceeding with a GA initially. A study comparing neonatal morbidity of emergency CD performed following the conversion of neuraxial anesthesia to GA with those performed under GA with no prior attempt to use neuraxial anesthesia found no difference in neonatal outcomes [16].

However, more extensive prospective studies are required to confirm this finding. Notably, our study found a 32% conversion rate from EA to GA. Conversion from EA to GA was not associated with worse neonatal outcomes, but our rate is higher than reported in the literature. Most studies have reported on the conversion rate for all categories of CDs, not solely category-1 [17]. In a study by Kinsella, conversion rates of EA to GA in category-1 CDs were found to be 20%, which was much higher than in cases with less urgency [18]. There appears to be an association between the urgency of CD and conversion from EA to GA, but there are other factors that influence the successful initiation of epidural surgical anesthesia that were not accounted for in our data. This includes active management of labor epidural analgesia with prompt replacement of poorly functioning catheters and close communication with the obstetric team. This allows the anesthesia team to optimize block density in preparation for CD and initiate conversion to EA as early as possible [19].

The overall rate of GA use for category-1 CD at our institution is slightly lower than that reported in the literature for an obstetric unit with a similar delivery volume [20]. This is likely due to a labor epidural analgesia utilization rate of > 75%, which has been shown to reduce the odds of requiring GA for emergency CDs [21].

The limitations of our study include the retrospective design, which is subject to selection and information bias. A CD initially designated as a category-1 could likely have been downgraded to a category-2 on arrival in the operating room, allowing adequate time to achieve surgical anesthesia using the epidural catheter. These scenarios are often chaotic, and the downgrading of the level of urgency may not always be documented accurately in the medical records. Umbilical arterial blood gas data were unavailable for thirty-one women, which could have potentially introduced bias to our outcomes. Additionally, the study consisted of a relatively small sample size and an uneven distribution between GA and EA groups. Future prospective multi-center studies may overcome these limitations. Finally, considering that we offer CSE for labor analgesia to most of our parturients, the findings in this study may not be generalizable to parturients whose labor epidural analgesia is not initiated with a CSE technique.

## Conclusions

Our results suggest that the conversion of epidural analgesia to surgical anesthesia for category-1 CD in women with a functional labor epidural catheter is not associated with poorer neonatal outcomes compared to category-1 CD done under GA. This result further supports the early placement of epidural catheters for labor analgesia, especially in parturients at increased risk of emergent CD.
